# Large-cage assessment of a transgenic sex-ratio distortion strain on populations of an African malaria vector

**DOI:** 10.1186/s13071-019-3289-y

**Published:** 2019-02-06

**Authors:** Luca Facchinelli, Ace R. North, C. Matilda Collins, Miriam Menichelli, Tania Persampieri, Alessandro Bucci, Roberta Spaccapelo, Andrea Crisanti, Mark Q. Benedict

**Affiliations:** 1 Department of Experimental Medicine, University of Perugia, Perugia, Italy; 2 Present address: Department of Vector Biology, Liverpool School of Tropical Medicine, Pembroke Place, Liverpool, L3 5QA UK; 3 Department of Zoology, University of Oxford, New Radcliffe House, Woodstock Road, Oxford, OX2 6GG UK; 4 Centre for Environmental Policy, Imperial College London, 16-18 Princes Gardens, London, SW7 1NE UK; 5 Polo di Genomica Genetica e Biologia, Via mazzieri 3, 05100 Terni, Italy; 6 Department of Experimental Medicine, University of Perugia, 06132 Perugia, Italy; 7 Department of Life Sciences, Imperial College London, Sir Alexander Fleming Building Imperial College Road South Kensington, London, SW7 2AZ UK; 8 Centers for Disease Control and Prevention (CDC), 1600 Clifton Road, Atlanta, GA 30329 USA

**Keywords:** *Anopheles gambiae*, Genetic control, Male-bias, Genetic load, Population model, Validation, Malaria

## Abstract

**Background:**

Novel transgenic mosquito control methods require progressively more realistic evaluation. The goal of this study was to determine the effect of a transgene that causes a male-bias sex ratio on *Anopheles gambiae* target populations in large insectary cages.

**Methods:**

Life history characteristics of *Anopheles gambiae* wild type and Ag(PMB)1 (aka ^gfp^124L-2) transgenic mosquitoes, whose progeny are 95% male, were measured in order to parameterize predictive population models. Ag(PMB)1 males were then introduced at two ratios into large insectary cages containing target wild type populations with stable age distributions and densities. The predicted proportion of females and those observed in the large cages were compared. A related model was then used to predict effects of male releases on wild mosquitoes in a west African village.

**Results:**

The frequency of transgenic mosquitoes in target populations reached an average of 0.44 ± 0.02 and 0.56 ± 0.02 after 6 weeks in the 1:1 and in the 3:1 release ratio treatments (transgenic male:wild male) respectively. Transgenic males caused sex-ratio distortion of 73% and 80% males in the 1:1 and 3:1 treatments, respectively. The number of eggs laid in the transgenic treatments declined as the experiment progressed, with a steeper decline in the 3:1 than in the 1:1 releases. The results of the experiment are partially consistent with predictions of the model; effect size and variability did not conform to the model in two out of three trials, effect size was over-estimated by the model and variability was greater than anticipated, possibly because of sampling effects in restocking. The model estimating the effects of hypothetical releases on the mosquito population of a West African village demonstrated that releases could significantly reduce the number of females in the wild population. The interval of releases is not expected to have a strong effect.

**Conclusions:**

The biological data produced to parameterize the model, the model itself, and the results of the experiments are components of a system to evaluate and predict the performance of transgenic mosquitoes. Together these suggest that the Ag(PMB)1 strain has the potential to be useful for reversible population suppression while this novel field develops.

## Background

Many bloodsucking arthropods are efficient vectors of pathogens responsible for human diseases worldwide [1]. Their genetic manipulation as a promising tool to control vector-borne diseases is promoted by the lack of vaccines for the majority of the infections they transmit, the spread of insecticide and drug resistance [2], and the expense of the vector control methods currently in place. Malaria is the arthropod-borne disease that causes the most mortality and morbidity and is transmitted by *Anopheles* mosquitoes primarily in tropical and sub-tropical environments [3]. Population suppression and population replacement [4] are two transgenic mosquito strategies that are being proposed to complement current malaria control methods, and in the past decade researchers have engineered *Anopheles* spp. strains able to block parasite development [5, 6] or bearing genes for population control [7, 8]. To be effective at low cost, these strategies must rely on transgenes able to spread through target populations with a super-Mendelian inheritance i.e. ‘gene-drive’ systems [9, 10]. This is predicted to result in a high transgene prevalence with relatively small field releases [11, 12].

The development of CRISPR-Cas9 technology recently allowed modification of the genomes of *Anopheles stephensi* [13] and *Anopheles gambiae* [14, 15] with transgenic constructs that are able to spread (drive) in target populations conferring resistance to *Plasmodium falciparum* infection or targeting female fertility genes. These proofs of principle for malaria control are tremendously promising, but a gap between the laboratory development and the field deployment of such technology exists, consisting in part of validated models that accurately predict the effects of transgenic insects in natural environments. The advances in driving transgenes represent a potential environmental and security concern [16], and researchers must demonstrate that their products can predictably and successfully reduce disease transmission and are safe for humans and the environment.

In order to obtain public and regulatory acceptance, the lab-to-field transition of transgenic mosquito strains is a multi-disciplinary, multi-step process, the final goal of which is to prove its reliability in terms of effectiveness, safety, and feasibility for field deployment. An important part of the transition includes testing in appropriate contained conditions that include increasingly realistic environments and the testing of strains that have less powerful capabilities than driving transgenes to minimize their spread when tested in endemic countries. These may have less effect or be more technically demanding to deploy [17].

The predicted benefit of genetic interventions is generally based on models of varying sophistication, ranging from simple algebraic calculations [18] to spatially explicit elaborate mathematical models [19]. In some cases, models are developed as an aid to design experiments [20] and in others the model is developed and tested in simplified laboratory experiments by comparing their predictions with actual outcomes [21]. Differences between predictions and outcomes are an indication of the value of models since they can identify parameterization errors, due for example to differences between parameter values in small and large cages, or model over-simplification. An iterative process of model refinement and testing is recommended as a process to develop models that are useful for planning interventions that will be implemented in natural environments [17]. This process is an essential part of evaluation because the effects of variation in parameter estimation are naturally greater when extrapolated to larger scales of testing.

Because male mosquitoes neither transmit pathogens nor feed on humans, they can be released safely to introduce heritable characters into wild populations. Causing female infertility by inundation with sexually sterile males (Sterile Insect Technique, SIT) is a proven and widely used form of genetic control, but other methods have been proposed using transgenic insects. Among these are forms that bias the sex ratio toward males [7]. One such strain is the transgenic Ag(PMB)1 (aka ^gfp^124L-2) strain which is characterized by 95% male-bias among the progeny of transgenic males (but not females) with no reduction in the number of eggs produced by female mates nor in the egg-hatching rate [7]. Male-bias in this strain is achieved by expression of a modified I-PpoI nuclease in the testes that cuts its 15 bp target site in the ribosomal DNA resulting in chromosome breakage which, in *An. gambiae,* is generally located solely on the X chromosome [22]. Therefore, expression of I-PpoI results in a majority of the sperm in Ag(PMB)1 males carrying only a Y chromosome. Because female sex in *Anopheles* spp. is determined by an XX karyotype and maleness by an XY karyotype [23, 24], matings by Ag(PMB)1 males in which the X chromosome has been cut result mostly in sperm carrying Y chromosomes and consequently a large majority of male progeny, half of which are transgenic.

As part of the progressive evaluation of a male-bias strain, large cages studies were performed to determine the effect of regular releases of hemizygous Ag(PMB)1 males on stable age-distribution *An. gambiae* populations. The resulting data were compared with a model that predicted the outcomes of the large cage studies. Subsequently, a field-informed model was applied to determine the effect of releases on mosquito populations in a West African village.

## Methods

### Mosquito strains

Two strains of *An. gambiae* were used for these studies: the transgenic ^gfp^124L-2 strain and the ‘wild-type’ G3 strain (MRA-112, Malaria Research and Reference Reagent Resource Center, Manassas, VA, USA). The G3 strain originated in The Gambia in 1975. The life history data reported and cited in this manuscript were all measured in this genetic background. The area where this strain originates is known to contain high levels of hybridization between *A. coluzzii* and *A. gambiae* but the MR4 reports that their holding consists only of *A. gambiae* rDNA. While this is an old laboratory strain, it has been demonstrated to have maintained at least one natural characteristic, male swarming [25]. The transgenic strain has been renamed ‘Ag(PMB)1’ to reflect the Paternal Male Bias phenotype by the organization that supported its development, Target Malaria [7], and this name is used hereafter in this paper. Ag(PMB)1 was created by genetic transformation of the G3 strain and it was maintained by crossing either transgenic males or females to G3 resulting in the two strains having the same genetic background. The 3XP3-DsRed transgene marker is visible in the thoracic and abdominal ganglia and the optic lobes. Backcrossing is performed to avoid the accumulation of rDNA damage that might occur in inbred strains. The G3 strain was also used as the experimental target population.

### Baseline measures of life history and mating competitiveness

In order to parameterize the model, life table studies were performed to compare the transgenic with non-transgenic sibling individuals of both sexes for four characteristics: (i) mortality during the larval stage; (ii) duration of the larval stage; (iii) pupa mortality; and (iv) adult survival. Throughout this paper, the terms ‘non-transgenic’ is used interchangeably with ‘wild-type’ in the context of laboratory studies. This equality reflects in part an assumption we made in this design, particularly that the extensive backcrossing (> 170 generations) used to maintain this strain is believed to have resulted in near genetic and life history identity between the G3 strain and the non-transgenic progeny of hemizygous individuals.

For life table studies of the immature stages, a 17.5 cm cube cage was populated with 400 virgin adults (200 Ag(PMB)1 males and 200 G3 females). Females were offered a blood meal for 45 min as described below and their eggs were collected, hatched and first stage larvae separated according to the fluorescent marker using a Complex Object Parametric Analyzer and Sorter (COPAS, Union Biometrica, Boston MA, USA) which separates larvae based on the fluorescent transgene marker. Eight trays of 250 larvae were established, four with transgenic larvae and four with non-transgenic larvae. Larval development time, larval survival, pupal survival and eclosion were recorded.

Larval survival was calculated from the starting number of first-stage larvae (L1) and those that pupated. Pupal survival was calculated by the number of pupae that eclosed. Larval and pupal mortality were analysed using quasibinomial generalised linear models with the replicate (four trays for each larval type) fit as a block to account for the within-tray pseudoreplication inherent to these data. Mosquito type (transgenic status) was the main effect during the larval stage and the influence of both sex and transgenic status were evaluated at the pupal stage. Analysis of larval duration also used a quasibinomial generalised linear model to assess the influence of mosquito type and sex on the length in days of the larval stage. In all cases, the influence of main effects was assessed by stepwise deletion testing.

For adult longevity studies, transgenic individuals were distinguished visually in the fourth (final) larval stage using an Olympus BX7 stereomicroscope equipped with DsRed filters (Chroma, Bellows Falls VT, USA) and an X-Cite 120Q illuminator (Excelitas, Waltham MA USA). Transgenic males and females were produced by crossing Ag(PMB)1 transgenic females to G3 males. Twelve cages of the design of Savage & Lowe [26] were populated with 30 male and 30 female pupae that were either transgenic or not in all combinations with three replicates of each. Adults that did not eclose were replaced on the following day with adults from cages set aside for this purpose. A 10% sucrose solution containing 0.1% methylparaben added as a preservative [27] was provided for adults and renewed on a weekly basis. Caged adults were checked daily and dead specimens were counted and removed until almost all adults were dead (42 d) after which those remaining were counted.

The data arising from the experiments are a daily-interval time series for each of 12 cages. To allow for the temporal pseudoreplication arising from repeated measurement of sequentially-linked cohorts (replicates), mixed effects models were used to identify whether there was a significant effect on the proportion surviving over time as a function of mosquito sex, transgenic status and whether the other sex they were caged with was itself transgenic. Random effects were used to represent the pseudoreplication of within-cage trajectories. The survival was compared over the 42 days of the experiment and assessment of the main effects and their interactions was by model simplification using L-Ratio tests at *P* < 0.01 to avoid over-interpretation, with Akaike information criterion (AIC) comparisons to evaluate model fit. Mixed effects models here and elsewhere used the *nlme* package [28]. Throughout, statistical analyses were performed using R 3.4.1 [29].

The ability of similar-aged males to compete for virgin females was determined. Ag(PMB)1 transgenic larvae and non-transgenic siblings were cultured according to a standard procedure [30]. Pupae were separated in three small cages according to sex and fluorescent marker in order to obtain virgin transgenic males, virgin wild type males and virgin wild type females. Three to four day-old virgin adults (50 transgenic males, 50 wild type males and 100 wild type females), were introduced into three 16 m^3^ cages provided with the visual stimuli inducing *An. gambiae* males to swarm [25] and which are described further below. Adults were provided with a 10% sucrose solution and allowed to mate. After 48 h these were collected, separated by sex and transgenic status and females were provided with a blood meal and put individually in oviposition cups. Paternity type was identified by offspring analysis for the fluorescent marker. Data were the number of females mated by either transgenic or non-transgenic individuals; a *χ*^2^ proportion test was used to determine whether this differed from an expectation of equal numbers.

### Large-cage experimental facilities

Three large cages (each *c.*16 m^3^) located in one insectary room were provided with visual stimuli to encourage *An. gambiae* males to swarm, the typical natural mating behaviour of this species. These cages, the lighting arrangement and cycle has been described in detail previously [25]. Insectary rooms were kept at a stable temperature and relative humidity (RH) of 27 ± 0.5 °C and 70 ± 5% RH. A stack of clay bricks (24 × 24 × 36 cm) in each cage was wetted with water daily and mosquitoes were seen to use it as a resting shelter. Three cups containing cotton and a 10% sucrose solution were used as sugar feeders in each cage. As sucrose has no fragrance, a spoonful of honey was added to each cup to attract mosquitoes to the sugar feeders. The cups hung from a cord at three distances from the entrance: near, mid and far. A pulley system allowed refreshing of the feeders on a weekly basis without entering the cages. Other objects and shelters were introduced into each cage to increase environmental heterogeneity: one Correx**®** equivalent black tunnel (60 × 60 × 40 cm, W × L × H); a vertical X-shaped structure consisting of one blue and one black 40 × 100 cm Correx**®** equivalent panel, was constructed by inserting one into the other by half-length slots in the centre of the short side to form the figure resting on the cage floor.

### Releases into the large cages

**Fig. 1:**
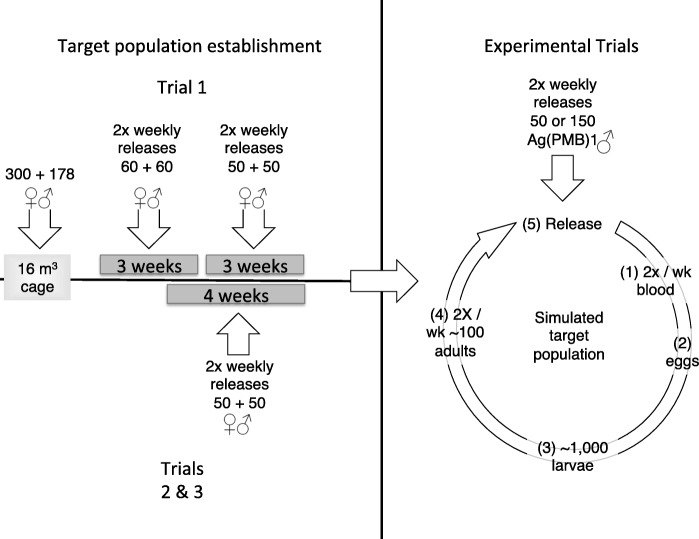
Schematic of large cage trials. Target populations were established by releasing equal numbers of G3 adult females and males into the 16 m^3^ cages for either 6 (Trial 1) or 4 weeks (Trials 2 and 3). In Trial 1, the target population was established with females and males from a preexisting stable age distribution population based on the model predictions of the population structure. In both Trials, after population establishment, semi-weekly releases of Ag(PMB)1 males were performed at two different ratios, or in the control, only progeny were returned to the control cage. (**1**) Mosquitoes were blood-fed using the artificial membrane feeder; (**2**) eggs were collected 3 days after the blood meal, bleached and incubated for 1 day; (**3**) 500 larvae were reared in two trays at a density of 250/liter/tray. When pupation started, immature stages from each tray were split in two trays and one of the four trays obtained was selected to be saved for restocking; (**4**) pupae were collected from the selected tray, sexed and screened for fluorescence before being divided according to sex in two small cages where adults could emerge and mature for 1–2 days; (**5**) twice a week virgin adults were introduced in the corresponding cage to maintain the population. At the same time, Ag(PMB)1 males the same age as the restocking adults were introduced into the treatment cages

Three sequential release trials were performed. In each, three cages were used: one control and two in which transgenic males were released at initial ratios of 1:1 or 3:1 (transgenic:wild type males) and which were expected to change as progeny of transgenic individuals appeared. Before starting the releases of transgenic mosquitoes into the experimental cages, G3 strain ‘target populations’ were established. The aim was to create populations that included individuals of all ages and mating status with a sex composition similar to a wild population. This was achieved by twice-weekly additions of G3 mosquitoes into each cage and allowing mortality and aging to stabilize the population. There were differences between the population establishment and duration of release observations between the first trial and the second and third (Fig. 1). In Trial 1, stable populations in the cages were established initially by adding 300 females and 178 males from a pre-existing stable G3 population (estimated sex ratio based on the model described below). After this, 60 G3 females and 60 males were added twice weekly for 3 weeks. After 3 weeks, 50 G3 females and 50 G3 males were added twice weekly for 3 weeks. After the experience of the first trial, the procedure for Trials 2 and 3 was altered slightly as the procedure for trial one was considered overly elaborate. Target G3 populations were established by introducing 50 females and 50 males twice-weekly for 4 weeks prior to releases. As the longevity studies indicated that adults live less than 6 weeks, these differences had negligible effect on the size of the populations when experimental releases began. However, to account for these differences when comparing the results to the model predictions, the model (described below) simulated each set of initial conditions separately.

The numbers of transgenic males released after the stable age populations had been established were either equal to (1:1) or three times (3:1) the numbers of G3 males being introduced previously, thus either 50 or 150 transgenic males twice-weekly. Mosquitoes added to the control cages were progeny only of that cage with no additional males added. During Trial 1, treatments were randomly assigned to each cage; during Trials 2 and 3, the treatments were rotated to different cages in order to minimize possible effects due to cage location in the environmental room.

To produce males for releases, Ag(PMB)1 transgenic males were crossed with G3 females. The life-cycles of mosquitoes used for release into the cages and those removed from the cages were maintained in synchrony so that similar-aged adults were available for releases and crossing. Larvae were reared using a slurry diet [31] using the method of Valerio et al. [30]. Ag(PMB)1 males used for cage releases were distinguished from non-transgenic individuals on the basis of the 3X-P3-DsRed fluorescent marker which was selected for using the COPAS in the first larval stage. The remaining *c.*5% females were removed manually by examining the terminalia under a stereomicroscope. In Trial 1, the introduction of Ag(PMB)1 males began 6 weeks after target population initiation and the experiment was terminated after 2 months. For Trials 2 and 3, releases began after 4 weeks of target population establishment and were terminated after 4 months.

During target population establishment and after transgenic mosquito releases began, females in the large cages were offered a blood meal on Monday and Friday at dusk for 2 h using a Hemotek membrane feeder (Discovery Workshops, Lancashire, England) containing sterile cow blood (Allevamento Blood di Fiastra Maddalena, Teramo, Italy). Parafilm® was used as an artificial blood-feeding membrane and was rubbed on human skin before covering the feeders in order to increase its attractiveness. Eggs were collected on 16 cm diameter polystyrene Petri dishes containing a water-soaked sponge covered by a filter paper disk. The oviposition dish was placed at the entrance of the cage close to the resting shelter 2 days after the blood meal was provided. A dim light was directed on the dish to concentrate oviposition during the dark hours. Previous work established that in the absence of this, eggs were often found on the cage floor (which was reflective aluminium) rather than in the oviposition dish (data not shown). The number of eggs laid was determined the following day by digital analysis of the disks using the Egg-Counter v1.0 software [32]. Egg hatching rate was determined after 3 days by microscopic examination of samples of approximately 200 eggs.

Once transgenic mosquito releases began, mosquitoes came from two sources: (i) the transgenic males released to simulate a suppression programme; and (ii) the progeny of adults of each cage to maintain the effects on the target population. To obtain the latter, eggs collected from each cage were hatched in trays and from these, *c.*500 larvae were reared in two trays. The L3-L4 stage larvae (approximately 250) from one randomly selected tray were divided between two trays, one of which produced the adults for restocking the experimental cages while the second one was maintained as backup. In the restocking tray, sex and transgenic status were determined in the pupal stage by examination under the Olympus BX7 stereomicroscope equipped with GFP filters and sex-separated to emerge in separate cages. All 1–2 day-old virgin adults from these emergence cages were then introduced twice-weekly into each cage. As the absolute numbers of eggs laid remained high and was variable through the trials, no effort was made to adjust the number of adults returned to the cages to reflect the numbers of eggs oviposited.

### Evaluating the effects of the releases

Several outcomes were anticipated to vary as a result of Ag(PMB)1 male releases: the number of eggs produced; the egg hatching rate; the frequency of transgenic offspring; and the proportion of females among offspring. Variation in these over time as a function of treatment was analysed by using linear mixed-effects (lme) models with day within cage fit as random variables to allow for the pseudoreplication created by repeated measures from within each cage. The key explanatory variable was the ‘Treatment’, a factor with three levels (Control, 1:1 and 3:1), the effect of which was also assessed by sequential trials.

Similarly, mixed-effects statistical models were fit to the proportion of females predicted in the computer simulation models and observed in the twice-weekly samples. Both the simulation model predictions and the experimental data were largely sigmoidal as a function of time and a logistic term was used. Maximum likelihood methods were then used to compare sequential lme models with progressively simplified fixed effects and this allowed assessment of consistency/comparison within and between model runs and experimental data [33]. As the different initialisation led to slightly different models, Trial 1 was examined separately to Trials 2 and 3. In all cases, as sampling effects were likely to be present and considered to contribute to the variability of the data, a threshold of *P* < 0.01 was applied to identify systematic effects.

### Predicting the effects of releases on the proportion of females

We modelled the effect of releasing Ag(PMB)1 transgenic males into a population using an iterative simulation model of the large cage experiments, and a related model of a village population. Both models track through time the numbers of juveniles (categorised by age, genotype and sex), unmated adult females (by genotype), adult males (by age and genotype), and mated adult females (by age, her own genotype and her mate’s genotype). Juveniles are assumed to emerge as adults 10 days after their oviposition (if they survive this long), and unmated females are assumed to mate with a random male on the day of their emergence. Mated females lay a Poisson-distributed random number of viable eggs per day (with expectation 9), though oviposition timing is restricted in the cage model (see below). The numbers of each possible egg genotype are randomised using a multinomial distribution that depends on the parent genotypes (assuming Mendelian inheritance). The sexes of the eggs are binomially-distributed, with male probability 0.95 if the father is hemi- or homozygous Ag(PMB)1 and 0.5 otherwise.

### Cage model

The cage model simulated oviposition only on 2 days/week (corresponding to Mondays and Thursdays), of which a random sample of 100 eggs is kept to become juveniles. All juveniles survive to be ‘added’ to the adult population 10 days after their oviposition. To simulate the treatment cages, zero-age Ag(PMB)1 hemizygous males were also added to the adult population on Tuesdays and Fridays after the treatment begun. Adult males and mated females have a Weibull-distributed randomised life-span, with Weibull shape and scale parameters fitted from the survival experiments. We simulated this model following the precise initial conditions of each replicate of the cage experiment.

### Village population model

The structure of the village model follows that of North & Godfray [34], except that here we consider only a single population rather than multiple connected populations. While the cage model does not consider juvenile mortality, the village model assumes juveniles suffer mortality due to both density independent causes (with probability 0.05 per day, estimated from measurements of larval survival when larval density is low ([35, 36]; see [37] for details), and from competition which varies with rainfall and local standing water [34]. We suppose competition mortality risk per day is $\sqrt[10]{\alpha (t)/\left(\alpha (t)+{J}_T\right)}$ where *J*_*T*_ is the total number of juveniles in the population, and *α*(*t*) controls the strength of density-dependent competition and is approximately proportional to the population carrying capacity. Specifically, *α*(*t*) is the number of juveniles at which the probability of death from larval competition over the course of development is 0.5, and we assume this variable depends on rainfall and the length of water courses in the vicinity of a population [34]. The time-dependence of *α*(*t*) stems from the input (weekly) rainfall data, and results in large seasonal population fluctuations for the West African setting we use (see below). This contrasts with the cage model for which population size is approximately constant in time. The village population model also differs from the cage model by assuming that adult males and mated females have constant daily survival probability, reflecting the numerous causes of mortality in an outdoor population which occur largely independently of age. Adult male daily survival was estimated to be in the range 0.69–0.87 (mean 0.77) from four mark-release-recapture experiments that took place in the village of Bana in Houet, South-West Burkina Faso [37], which we use as our study location. We use both the lower and upper of these estimates to investigate this parameter, and we set female survival at the somewhat higher value of 0.875 which is consistent with endemic malaria transmission.

The remaining parameters of this model arise from long-term studies in Bana, and are set to correspond with seasonal variations in population size determined from the same MRR experiments used to estimate mortality [37]. For the purpose of this analysis, we assume there is no migration in or out of Bana. This model uses the rainfall data from the ERA-interim reanalysis [38], and the water course data from the Digital Chart of the World (DCW) (available from http://www.diva-gis.org/Data), as described by [34].

The village population was simulated for 2 years prior to transgene releases, which was enough time to minimise effects of initialising the model with an arbitrary population structure. Transgene releases were simulated in the third year by adding various numbers of Ag(PMB)1 hemizygous males to the population at regular time intervals. Rather than attempting to simulate fixed release ratios such as were used to initiate the cage populations, these release numbers were based on discussions of potential production levels for existing and possible future insectary infrastructure.

## Results

### Baseline life history

In order to parametrise the predictive models, life table studies were performed to estimate any differences between Ag(PMB)1 transgenic individuals and non-transgenic siblings that would need to be taken into consideration. Larval mortality was 8.5% and did not vary as a function of transgenic status (*F*_(6,7)_ = 0.18, *P* = 0.69).

There was no identifiable effect of the transgene (*F*_(8,9)_ = 1.19, *P* = 0.31) or sex (*F*_(8,9)_ = 3.85, *P* = 0.09) on the duration of the larval stage (median 7 days). Whilst there was some mortality as pupae (6.6 ± 4.3%), this also did not vary as a function of sex (*F*_(12,13)_ = 1.21, *P* = 0.29) or transgenic status (*F*_(13,14)_ = 0.84, *P* = 0.37).

**Fig. 2:**
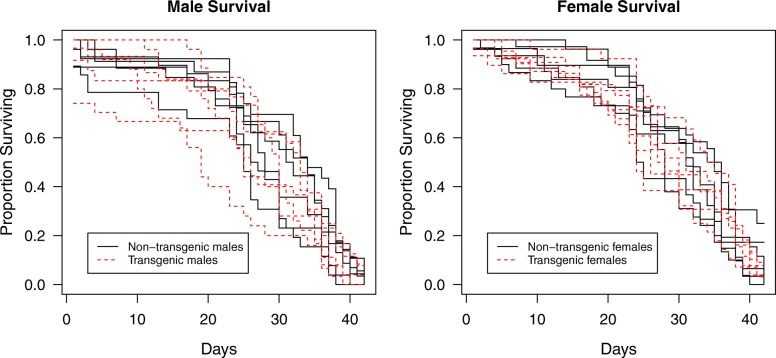
Adult survival. Proportion of survival of positive (transgenic) and negative (non-transgenic) adults through time (days after cage initiation)

As all combinations of males and females according to transgenic status were used, it was possible to determine whether there was an effect of the combinations on longevity. There were no interactions between the main effects, nor did the transgenic status of the mosquitoes or that of the accompanying mosquitoes affect their survival. The males and females did have different survival (L.Ratio_(6,7)_ = 25.00, *P* < 0.001); females lived slightly longer than males (median longevity 30 *vs* 28 days, Fig. 2).

Mating competitiveness was expected to be a critical factor for predictions of the transgene behaviour. This reflects the ability of one type of male to compete with another for matings with virgin females. Here, all males were of similar ages and of 176 mated females whose progeny were assessed for the transgene, Ag(PMB)1 achieved 54% of matings in competition with G3 males; this did not differ from an assumption of equal competitiveness (*χ*^2^ = 0.4, *df* = 1, *P* = 0.52).

### The effect of releases

#### The frequency of transgenic offspring in cage populations

Six weeks after the trials had started (from day 45 of measures), the frequency of transgenic mosquitoes in the target populations reached averages of 0.44 ± 0.02 and 0.56 ± 0.02 in the 1:1 and in the 3:1 initial release ratio treatments (transgenic male:wild male) respectively. The frequencies reached did not differ among trials (L.Ratio_(7,9)_ = 2.37, *P* = 0.30) or rise systematically with ongoing releases (L.Ratio_(6,7)_ = 1.14, *P* = 0.28), but was higher at the higher release rate (L.Ratio_(5,6)_ = 24.39, *P* < 0.001).

#### The effect on the proportion of females

Both release levels differed from the control (L.Ratio_(5,7)_ = 32.62, *P* < 0.001) in the proportion of females in the offspring after day 45, and the 3:1 treatment resulted in a lower proportion of females than the 1:1 treatment (L.Ratio_(6,7)_ = 19.55, *P* < 0.001, Table 1). Although the difference between the two treatment levels is statistically significant, the effect size is small.

**Table 1:** Effect of transgenic male releases on the proportion of females

	Proportion female after day 45 (Mean ± SE)	Difference between control and release cages	
		1:1	3:1
Control	0.49 ± 0.01	-0.22 ± 0.00	-0.29 ± 0.00
1:1	0.27 ± 0.01		-0.07 ± 0.00
3:1	0.20 ± 0.01		

#### Effects on egg production

**Fig. 3:**
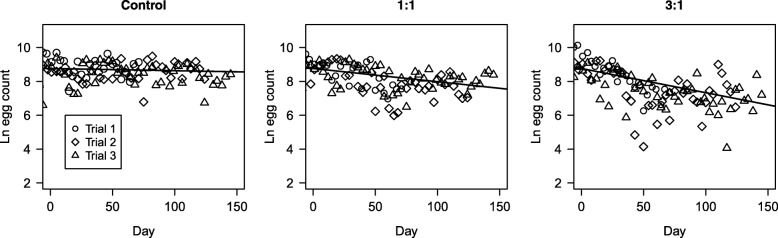
Number of eggs obtained during trials. The numbers of eggs that were laid as a function of the three treatments and trials through time (days into experiment). Lines represent the least-squares linear regression of samples

Because the proportion and number of females in the population were predicted to be reduced by the releases, the numbers of eggs produced as a function of time might also decline. There was no significant variation in egg production pattern between the three sequential trials (L.Ratio_(13,15)_ = 3.31, *P* = 0.19). Treatment did have a significant effect (Fig. 3). Egg production remained stable in the controls, but declined in both release treatments (L.Ratio_(10,13)_ = 30.42, *P* < 0.001). The decline was steeper in the 3:1 treatments than in the 1:1 (L.Ratio_(11,13)_=17.58, *P* < 0.001).

#### Effects on egg hatching rate

**Fig. 4:**
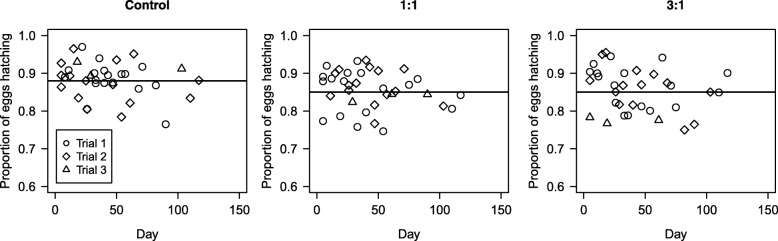
Proportion of eggs hatching. The proportions of eggs that hatched for the three treatments during the three trials. Lines represent the least-squares linear regression of samples

It was also anticipated that the releases could lead to additional mutations to the rDNA that did not result in broken X chromosomes that could accumulate in the population, possibly causing semi-sterility [7]. This could result from transmission of the possibly damaged X chromosome introduced into the population by the approximately 5% of female progeny that result from transgenic males. Therefore, to estimate this, the egg-hatching rate was determined. An average of 211 ± 3 eggs was assessed in each sample. There was no variation in the proportion of eggs hatching between the trials (L.Ratio_(13,15)_ = 0.07, *P* = 0.96, Fig. 4). The proportion hatching was the same in the 1:1 and 3:1 treatments (L.Ratio_(11,13)_ = 0.08, *P* = 0.96), but was slightly lower in these than in the control (0.86 ± 0.01 *vs* 0.88 ± 0.01) (L.Ratio_(9,10)_ = 8.13, *P* = 0.004). Hatching rate did not decline further during the experiment in any treatment (L.Ratio_(9,10)_ = 1.75, *P* = 0.18).

### Effects of releases on proportions of females *vs* model predictions

**Fig. 5:**
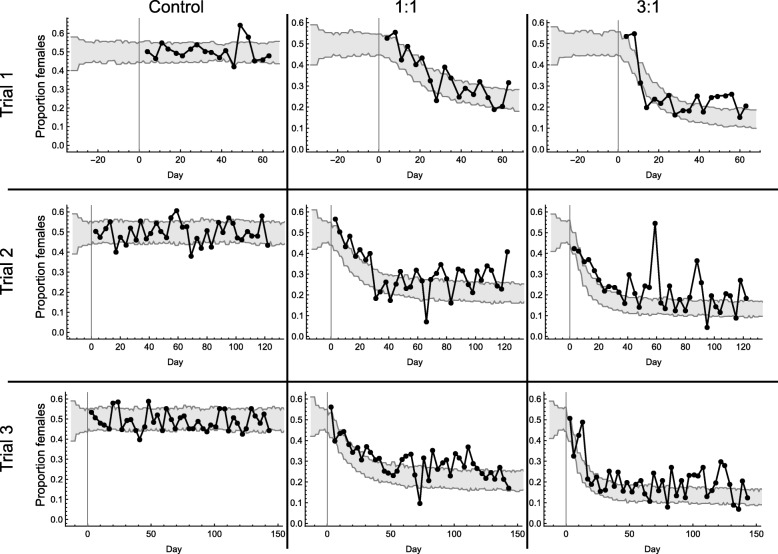
Observed proportion of females among progeny during trials. Grey bands represent the 95% CI for 100 model simulations with the observed values for the three trials overlain. The vertical lines represent the time at which releases began

The results of Trials 1–3 are shown in more detail in Fig 5. In this figure, we represent the predicted female frequency determined by the model (95% CI) with the observed female frequencies overlaid. In Trial 1, the proportion of females recorded in all treatments was consistent with the model predictions (Control: L.Ratio_(5,6)_ = 0.68, *P* = 0.41; Equal: L.Ratio_(6,7)_ = 0.37, *P* = 0.54; High: L.Ratio_(6,7)_ = 0.043, *P* = 0.84).

In the control cages of Trials 2 and 3, the proportion of females did not differ from each other (L.Ratio_(6,7)_ = 0.16, *P* = 0.69) nor did either trial differ from the model predictions (Trial 2: L.Ratio_(6,7)_ = 0.34, *P* = 0.56; Trial 3: L.Ratio_(6,7)_ = 3.35, *P* = 0.07).

In the 1:1 release ratio, Trials 2 and 3 did not differ from each other in the proportion females identified in the samples ( L.Ratio_(7,8)_ = 0.06, *P* = 0.79), but both do differ from the model predictions (Trial 2: L.Ratio_(7,8)_ = 20.62, *P* < 0.001; Trail 3: L.Ratio_(7,8)_ = 20.07, *P* < 0.001). In both these trials the proportion of females found is often above those predicted by the model.

In the 3:1 treatment, the two trials did not differ from each other in the proportion female found (L.Ratio_(7,8)_ = 0.55, *P* = 0.49). Both Trials 2 and 3 did differ from the model predictions (Trial 2: L.Ratio_(7,8)_ = 35.53, *P* < 0.001; Trial 3: L.Ratio_(7,8)_ = 31.02, *P* < 0.001), they were often both higher than predictions and displayed greater variability.

### Effects of hypothetical releases on the mosquito population of a village in West Africa

**Fig. 6:**
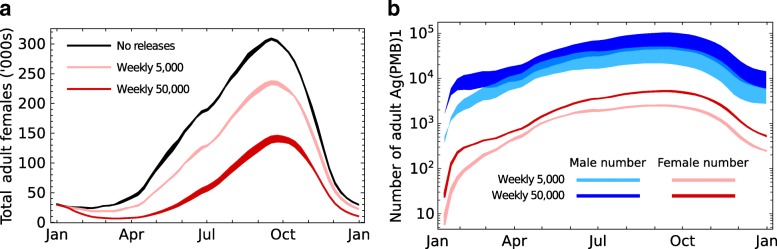
Effect of releases of Ag(PMB)1 males in a village. The effects of weekly releases of 5 or 50 thousand transgenic males compared with the expected number in a village in which there were no releases. **a** The effect on the number of females in the population. Y axis values are in thousands. **b** The number of transgenic females and males in the population. Two values of male daily survival were used to create the models, reflecting upper (upper lines; 0.87) and lower (lower lines; 0.69) estimates of MRR experiments [37] Epopa 2017 values

Regular releases of Ag(PMB)1 males into wild populations are predicted to have significant suppression effects on the female population of the species that is released (Fig. 6a) and predicts that large numbers of individuals carrying the transgene will exist in the population at the end of the calendar year which occurs during the dry season (Fig. 6b).

**Fig. 7:**
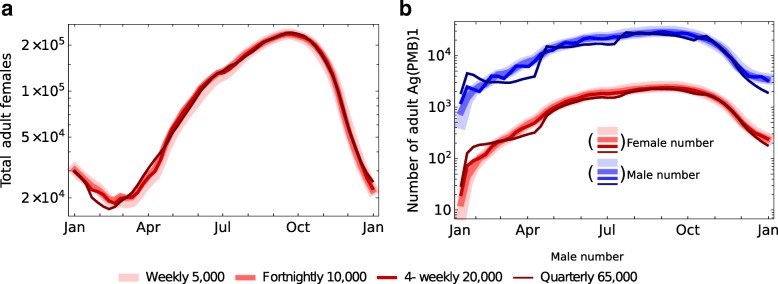
Effect of release frequency on the female population size and number of transgenic adults in a village population. Release frequency has little effect on the number of females in the target population (**a**) or the number of transgenic individuals (**b**) provided the total number released remains the same. We set male daily survival at the mean estimate, 0.77 [37]

This leads to the question whether release frequency, in contrast to numbers, is an important operational consideration for the use of this transgenic technology. Releases at any frequency less than quarterly are predicted by the model to have negligible differences in effect as long as the cumulative numbers released remain the same (Fig. 7).

**Fig. 8:**
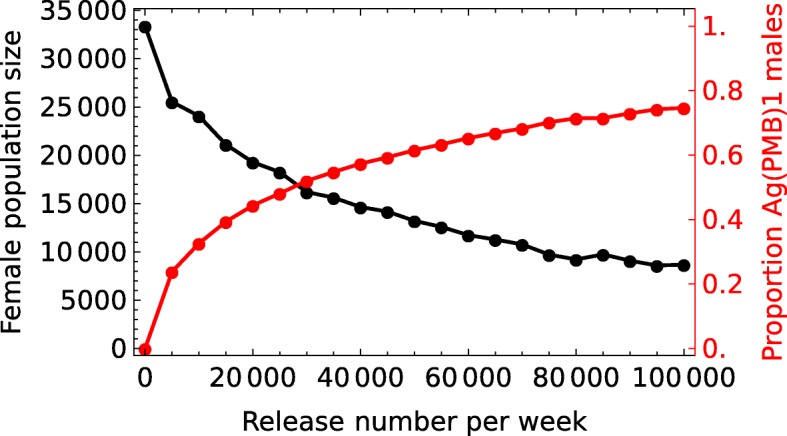
Female population size as a function of release numbers. Influence of weekly release size (various numbers of transgenic males up to 100,000) on the predicted female population size at the end of the calendar year. As in Fig. 7, male daily survival is set at 0.77

The relationship of increasing release size to the number of females at the end of the rainy season deserves further discussion since it has a direct impact on the cost-benefit and likelihood of detecting an effect of conducting a suppression program with strains such as this. Considering the example village, Bana, in which the unperturbed population size at the end of the rainy season is in the region of 35,500, weekly releases of 5000 would reduce the number of females by an estimated 25% to *c.*27,000 (Fig 8). Increasing the male-release size 10-fold to 50,000 is estimated to only decrease the predicted population a further 37% to *c.*13,500 females.

## Discussion

The release of transgenic mosquitoes as a novel control method should be developed in a progressive manner starting with types of transgenic strains whose effects and persistence will be limited [17]. This has been proposed as a means of technology implementation that best results in environmentally safe programs with predictable effects. We propose that Ag(PMB)1 might in part serve this purpose. Ag(PMB)1 has a useful phenotype whose life-history traits in controlled large cages trials demonstrate no negative effect of the transgene that would make it a poor candidate for release. Unlike a transgenic sterile male strain with which we have previous similar experience [21], few differences between model predictions for cages and the experimental outcomes were observed. In the case of the sterile males, the degree of difference between the model prediction and transgene frequency indicated that there were significant fitness costs associated with the transgene that were not apparent from life-history studies.

Even if only modest numbers of Ag(PMB)1 male mosquitoes were released that did not result in measurable reductions in females, any persistence observed in natural populations would provide a valuable description of the behaviour of this transgene that could, in turn, be compared with model predictions. Identifying discrepancies between the model predictions and observations would provide further insight into life history parameters that may have been inadequately understood in the previous underpinning studies.

Generally, differences between outcomes of studies conducted at different scales can be attributed to two causes: inaccurate estimates of parameters due to sampling error and differences in parameters due to differences in stressors, complexity and scale that affect the biology of the organism. The latter are more problematic since merely increasing the number of trials in the laboratory cannot improve the estimates. In recognition of the latter, laboratory estimates of adult survival were used for the cage simulation model whereas field estimates of male daily mortality were used for the population model. Extrapolating the laboratory parameter would be highly misleading. Other values such as mating competitiveness were not available for natural populations but could reasonably expected to differ from the laboratory studies. Sometimes, there is an experimental basis for doubting extrapolation of small cage studies to natural populations; Facchinellli et al. [25] demonstrated that male competitiveness of a transgenic sterile-male strain was negatively affected by larger cage size, a trend which could reasonably be expected to continue to natural populations. There was no basis on which to expect such a difference for the Ag(PMB)1 studies.

Therefore, an essential value of iterative testing, parameterization and prediction is to arrive at more realistic estimates of the parameters that are finally used to model population effects. Field studies of transgenic mosquito interventions provide the most rigorous test of parameters and models but these can only be approached cautiously based on the best information available. This process also highlights the value of realistic indoor and outdoor contained studies of population simulations.

The large cage data observed here indicated that the village population model might be optimistic in its predictions of the extent of observable suppression as the proportion of females found in the large cage studies of Ag(PMB)1 was slightly higher than the intervals predicted by the model. Estimating the size of subtle differences between data and model can be challenging in the light of high-variance in samples (see below). These observations do largely support the predictions that significant effects might be observed in field populations with the release of only modest numbers of adults compared to ‘sterile insect technique’ types of programs in which e.g. 10-fold inundation is often considered a minimum to obtain an effect [40].

This difference from the release of sterile males is due in part to the fact that selection against the Ag(PMB)1 transgene is much weaker than that of either males that are sexually sterile or which confer lethality to their progeny against which selection is acute and final. The resulting accumulation of transgenic females and males and persistent phenotypic effect of the transgene in target populations and the lack of sensitivity to release frequency should permit greater flexibility than SIT which is sensitive to release frequency [19]. When releases of sterile males stop, the population immediately begins to rebound. That is not anticipated to be the case with male-bias strains such as the example that was studied here. Therefore, suppression effects should be relatively resilient to interruptions in releases due to e.g. bad weather, transportation difficulties and production-level fluctuations. Natural *Anopheles* populations in Burkina Faso are often a seasonally-dependent mixture of three members of the *An. gambiae* complex [39], and we considered only one species in the simulation village, Bana; therefore the model demonstrates the effect only on the species that is released, a factor that would need to be considered when predicting possible epidemiological effects.

Experimental design has a decisive effect on the outcome of simulations such as those which were conducted here. The outcomes of these large cage studies contrast with the results observed by release of a similar male-bias strain in small cages [7] in which extinction of the target populations was usually observed within six generations and egg-hatching rates were only 20% in the terminal generations even though the transgenic male release rate was also 3:1. These differences can be attributed simply to two changes that were made in the experiments reported here: (i) a stable age distribution population was established before the introduction of transgenic males; and (ii) the populations were continuously breeding rather than consisting of discrete generations. This results in a more-realistic simulation of the conditions that occur in nature and reflects the stable equilibrium of the sex ratio that is expected to be reached in such caged populations. A natural effect that our experiments do not reflect is the potential reduction in the reproductive rate of the population that might occur due to a decline in the population rate of increase and the resulting increase in the effective transgenic male release rate. These effects are captured, however, in the village population model.

Another aspect of experimental design, the longer duration of Trials 2 and 3, enabled the identification of effects not apparent during Trial 1. Sampling variability was anticipated in all measures, for example, the egg sample could be affected by the number of ovipositing females, and thus their mating partners, laying eggs in an aggregated manner and affecting the arising measures. This potential ‘founder effect’ could then influence the measure of hatch rate or the proportion females identified in that time step sample. The longer runs thus give a clearer picture of both the sampling variability and the systematic effects, so whereas the shorter Trial 1 does not differ from model predictions, the difference from model predictions in Trials 2 and 3, though slight, is apparent by virtue of the length of the experiment. Identifying these systematic differences is vital to model validation and enables further refinements so that predictions at wider spatial scales will be more realistic. This may well call for the explicit inclusion of an element of sampling variability in predictive models to reduce the likelihood of variability in data masking general conformity to the predictions. In field trials the sampling effect is likely to be greater still and will depend on the availability and tractability of monitoring techniques. Many that are deployable at scale, such as ovitraps, are known to produce high deviance, overdispersed data [41, 42]. This will require careful thought as the field progresses and will be helped by trials designed to explicitly estimate sample deviance.

Producing male mosquitoes for release can be logistically challenging if the strains are not pure-breeding and there is no genetic method to eliminate females. For example, the ability to suppress an effector that is counter-productive to rearing - bi-sex lethality - has been essential for production of the *Aedes aegypti* OX513A strain which can be cultured in a pure-breeding colony in the presence of tetracycline [43]. Females of *Ae. aegypti* can also be eliminated based on pupa size [44], the combination of these two methods enables production of tens of millions of males [45]. The Ag(PMB)1 strain of *An. gambiae* does not have either characteristic: it is not pure-breeding and there is no *en masse* male selection method. However, generic means that are suitable for routine production of Ag(PMB)1 mosquitoes have been devised. Use of the high-throughput COPAS sorter to segregate transgenic mosquitoes [46] and a male-specific fluorescent marker [47] could provide a larval sorting method for both transgenic males to release and to select non-transgenic females for backcrossing to maintain the stock. Using the COPAS in this way could permit rearing of 50,000 larvae per week in compact facilities using high density larval rearing systems (e.g. [48]) and pupa selection [49].

Strains such as the one considered here have potential to contribute towards local suppression of females at a village-scale, though they do not appear feasible for area-wide suppression at a country or continental level. This is because of their limited persistence and production challenges, therefore for wider-scales, ‘gene-drive’ systems have been proposed as potentially effective [4, 10]. These would offer the potential to spread female infertility [14] or male-bias via ‘Y-drive’ [47, 50] and would need to be developed as technologies that could be deployed at feasible cost [15].

The insertion on the strain tested here is on an autosome [7], but if the transgene could be inserted and expressed from the Y chromosome it would be inherited by all male progeny, resulting in a ‘driving Y chromosome’ [47, 50] that is expected to increase in frequency and potentially result in population suppression. Reproducible insertion of genes on the *An. gambiae* Y chromosome has been accomplished [47], so two essential parts of such a system have been realised, the male-biasing transgene and modification of the Y chromosome.

The other aspect of the potential of these strains that takes full advantage of the limited persistence and incomplete eradication they offer is the stepwise development and evaluation of these transgenic technologies [17]. Local reductions would enable monitoring and assessment of non-target-organisms (NTOs) potentially affected by changes in the density of *An. gambiae*. There is an understanding of what potential interacting species there are [51] and key non-target organisms could be studied more specifically in tandem with releases to evaluate hypothesised effects.

## Conclusions

An integrated process of modelling, experimental trials, analysis and reparameterization must be conducted in progressively more realistic settings to arrive at predictions for field behaviour that approximate real field outcomes. The results presented here are an example of such a process and demonstrate that, to the degree tested, Ag(PMB)1 could be considered for field release as a female suppression technology. The insensitivity of suppression to release frequency makes this strain and others with high persistence and similarly weak negative selection attractive for release programs that might be interrupted by production shortfalls and other disruptions. Novel transgenic technologies are likely to require demonstrations of conformation to prediction, and the process and strain used here are a key part of developing confidence in this field.

## Abbreviations

AIC: Akaike information criterion
CI: confidence interval
COPAS: Complex Object Parametric Analyzer and Sorter
lme: linear mixed-effects
RH: relative humidity
SIT: sterile insect technique
